# Interferons in Sjögren’s Syndrome: Genes, Mechanisms, and Effects

**DOI:** 10.3389/fimmu.2013.00290

**Published:** 2013-09-20

**Authors:** He Li, John A. Ice, Christopher J. Lessard, Kathy L. Sivils

**Affiliations:** ^1^Arthritis and Clinical Immunology Research Program, Oklahoma Medical Research Foundation, Oklahoma City, OK, USA; ^2^Department of Pathology, University of Oklahoma Health Sciences Center, Oklahoma City, OK, USA

**Keywords:** interferon signature, Sjögren’s syndrome, gene expression profiling, microarrays, type I interferon, genetic association, mechanisms, biomarker

## Abstract

Sjögren’s syndrome (SS) is a common, progressive autoimmune exocrinopathy distinguished by dry eyes and mouth and affects ∼0.7% of the European population. Overexpression of transcripts induced by interferons (IFN), termed as an “IFN signature,” has been found in SS patients. Four microarray studies have been published in SS that identified dysregulated genes within type I IFN signaling in either salivary glands or peripheral blood of SS patients. The mechanism of this type I IFN activation is still obscure, but several possible explanations have been proposed, including virus infection-initiated and immune complex-initiated type I IFN production by plasmacytoid dendritic cells. Genetic predisposition to increased type I IFN signaling is supported by candidate gene studies showing evidence for association of variants within IFN-related genes. Once activated, IFN signaling may contribute to numerous aspects of SS pathophysiology, including lymphocyte infiltration into exocrine glands, autoantibody production, and glandular cell apoptosis. Thus, dysregulation of IFN pathways is an important feature that can be potentially used as a serum biomarker for diagnosis and targeting of new treatments in this complex autoimmune disease.

## Introduction

Sjögren’s syndrome (SS) is a chronic autoimmune disease that primarily affects middle-aged women with an estimated prevalence of ∼0.7% in European populations (1, 2). SS is characterized by infiltration of lymphocytes into glandular tissues, typically the salivary and lacrimal glands, leading to xerostomia (dry mouth) and keratoconjunctivitis sicca (dry eye). The resulting pathology can be debilitating, and target organ damage may be so severe that moisture production is virtually non-existent. However, manifestations of SS are not limited to exocrinopathy; other common extraglandular features include fatigue, arthritis, Raynaud’s phenomenon, and an increased incidence of non-Hodgkin B cell lymphoma (3). Two autoantibodies targeting ribonucleoproteins, anti-Ro/SSA, and anti-La/SSB, are detected in 60–70% of SS patients and are important to disease diagnosis (4, 5).

The etiology and pathogenesis of SS are still unclear, partially due to the complexity and heterogeneity of disease mechanisms. Recently, the dysregulation of interferon (IFN) signaling pathways, especially upregulation of type I IFN-inducible genes, has been observed in salivary glands and peripheral blood in a subset of SS patients (6– 10). Type I IFNs, including IFNα and IFNβ, are key immune mediators involved in viral defense and activation of immune responses (11). Viral infection has long been suspected to trigger SS (12), and abnormal elevations in type I IFN signaling may reflect an important role for viral infection in disease pathogenesis. Additionally, genetic association studies indicate the importance of multiple genetic loci within IFN pathways. Here, we review the identification of the type I IFN “signature” through high-throughput techniques, and discuss potential mechanisms and functions of dysregulated IFN signaling in SS.

## Identification of the “IFN Signature” in SS

Many powerful, high-throughput techniques have emerged in the last few decades and have revealed important insights into mechanisms of complex human diseases. Gene expression profiling (GEP) studies using microarrays represent one of the most widely used approaches to determine global transcriptome differences between patients and healthy controls. “Signatures” of disease have been defined that represent clusters of co-expressed genes, often within a biological network, that may serve as biomarkers for disease diagnosis, classification, and drug response prediction. The “IFN signature,” first described in a GEP study of systemic lupus erythematosus (SLE), has been defined by the overexpression of type I IFN-inducible genes (13). Subsequent studies have demonstrated similar signatures in other autoimmune diseases, such as rheumatoid arthritis (RA), systemic sclerosis (SSc), myositis, and SS (14).

Four microarray studies have been published in SS to date that describe the overexpression of type I IFN-inducible transcripts in minor salivary glands or peripheral blood from SS patients (7–10). Hjelmervik et al. (7) published the first GEP in SS using a relatively low-density 16K microarray to identify a whole transcriptome signature in biopsy samples of minor salivary glands from 10 SS patients and 10 controls who experienced subjective oral dryness. They successfully clustered 19 out of the 20 subjects into the correct group by using the top 200 differentially expressed transcripts, in which numerous type I IFN-regulated genes were represented, including *IFI27*, *ISG12*, *GBP2*, *IFITM1*, and *IRF8*. Subsequently, Gottenberg et al. (8) also identified distinct gene expression patterns involving IFN pathways (both type I and type II) in salivary glands of SS patients by comparing seven cases and seven controls. Specifically, 23 genes were IFN-inducible, including genes in the antiviral IFN-induced transmembrane protein (IFITM) family (*IFITM1*, *IFITM2*, and *IFITM3*) and genes in the Toll-like receptor (TLR) family (*TLR8* and *TLR9*) that play a fundamental role in pathogen recognition and activation of innate immunity (15). All showed significantly increased expression in SS patients. Interestingly, the only two known IFN-inducible genes that showed decreased expression in SS salivary glands, *CCL18* and *SOCS3*, are involved in the inhibition of inflammatory processes. The overexpression of IFN-inducible genes was supported by the detection of plasmacytoid dendritic cells (pDC), the most potent producer of type I IFNs (16), in salivary glands of patients with SS, but none in the glands of controls. These results suggest that pDC activation may play a role in SS pathogenesis, which is discussed later in this review.

Upregulation of 11 IFN-inducible genes has been identified by Pérez et al. (10) through a microarray study of epithelial cells from salivary glands of nine SS patients and six controls. Notably, three of these genes belong to the IFN regulatory factor (IRF) family (*IRF7*, *IRF8* or *ICSBP1*, and *IRF9*). IRFs are pivotal transcriptional regulators of type I IFN and IFN-inducible genes, and are important in cellular differentiation of hematopoietic cells (17). This GEP study also identified dysregulation of apoptotic pathways in SS epithelial cells, which are now thought to be involved in local auto-antigen production and tissue damage in the salivary glands of SS patients (18, 19). Additionally, they identified six genetic loci associated with SS using microsatellite markers, with five of the association signals falling within regions where differentially expressed genes were found, such as *IL6*, *CD44*, and *IRF9*. These results support a genetic contribution to the dysregulated IFN pathways observed in SS.

The IFN signature has also been observed in peripheral blood of SS patients. Emamian et al. (9) detected upregulation of IFN-inducible genes in peripheral blood of a subset of SS patients by comparing 21 cases and 23 controls followed by replication in an independent dataset of 17 SS cases and 22 controls. *IFI35*, *MX1*, *OAS1*, *IRF7*, and *OAS2* were among the top differentially expressed genes and are known to be induced by IFNs. The authors also showed that the expression levels of most IFN-inducible genes were positively correlated with anti-Ro/SSA and anti-La/SSB titers. Although the relationship between IFN pathway activation and autoantibody production is unclear, these results provide a link for both innate and adaptive immune responses to the pathogenesis of disease. These results also suggest that the IFN signature can be potentially used as a disease biomarker for a subgroup of SS patients with certain clinical features that includes the production of anti-Ro/SSA and anti-La/SSB.

All of these microarray studies have consistently identified differentially expressed genes in IFN-mediated signaling pathways. As shown in Table 1, there are several differentially expressed genes found to be common across multiple studies and multiple tissue types, such as *IFITM1*, *IFI44*, *MX1*, *IRF7*, and *IRF8*, suggesting both local and systemic dysregulation of IFN signaling pathways in SS patients (Table 1). Each study also revealed unique dysregulated genes, partially due to the different types of arrays, relatively limited sample sizes, different quality control processes, and sample heterogeneity between studies.

**Table 1:** **Differentially expressed IFN-inducible genes found in common from the gene expression profiling studies in SS**.

	Hjelmervik et al. (T score)	Gottenberg et al. (Fold change)	Pe?rez et al. (Mean difference with log_2_ scale)	Emamian et al. (Average fold change)	Gene function
*IFITM1*	−4.98	2.52	1.13	1.83	Block early stages of viral replication
*IFITM3*		1.88		1.96	
*IFIT2*			1.11	2.39	Inhibits expression of viral mRNA lacking 2′-O-methylation
*IRF7*			0.93	2.18	Important transcription regulators of type
*IRF8*	−4.40		1.47		I IFN and IFN-inducible genes
*IRF9*	−5.72		0.74		Important to cell differentiation
*IFI16*			0.99	1.56	Modulates p53 function and inhibits cell growth via Ras/Raf pathway
*IFI27*	−6.29			15.83	Mediates IFN-induced apoptosis
*IFI44*	−4.74			3.49	Antiproliferative, associated with HCV infection
*MX1*			1.15	3.85	A GTPase with antiviral activity against a wide range of RNA viruses and some DNA viruses
*SP110*	−5.05			1.85	Regulates gene transcription

Additional studies have identified increased activation of type I IFN-mediated genes in SS patients by candidate gene approaches (20–24). Increased IFN-inducible gene expression profiles have been detected in saliva and tears as well as particular cell types from SS patients (25, 26). However, results for the expression levels of IFNα itself in SS patients are controversial (6, 27). This may be due to the different techniques used in separate studies, a mixture of cell types in each experiment, and the heterogeneity of SS patients involved.

## Mechanisms of Elevated IFN-Mediated Signaling in SS

Although virtually all cells can produce type I IFNs in response to viral and bacterial infection, pDCs are the most potent IFN-producing cells, making up to 1000-fold more type I IFNs than other cell types (28). The detection of activated pDCs in salivary glands of SS patients but not in controls makes pDCs prominent candidates for the local production of type I IFNs that may promote the formation of inflammatory foci (8). Activated pDCs have also been found in the target organs of other autoimmune diseases (29, 30). Interestingly, Wildenberg et al. (26) found that, although the number of pDCs is decreased in the blood of SS patients, supposedly due to the migration of pDCs to peripheral sites, the cell surface activation marker CD40 is significantly overexpressed on pDCs from SS patients. Possible explanations of sustained activation of pDCs in SS include chronic exogenous stimulation and constitutive expression of pro-inflammatory transcription factors, such as IRF5 and IRF7, in SS patients (31).

Type I IFNs are induced transiently by viral infection and elicit antiviral effects. The pDCs can be rapidly induced to produce IFNα upon stimulation by RNA and DNA through TLR7 and TLR9, respectively (32). Thus, an initial viral infection is suspected to trigger the production of type I IFN by pDCs. The contribution of viral infection to elevated IFN signaling in SS is unknown; however, a number of viruses have been thought to contribute to SS pathogenesis, including Epstein–Barr virus (EBV), cytomegalovirus (CMV), hepatitis B virus (HBV), and hepatitis C virus (HCV) (12). Several mechanisms have been hypothesized regarding possible infectious triggers of SS, such as antigenic molecular mimicry. For example, infection with EBV results in the production of EBV nuclear antigen-1 (EBNA-1). Immune response against EBNA-1 can generate antibodies that cross-react with SS-associated autoantigens, such as anti-Ro/SSA (33). These antibodies may undergo epitope spreading and may ultimately become pathogenic in SS.

Autoantibodies and autoantigen-specific B cells have been detected in the salivary glands of SS patients (34, 35) and may involve in the production the type I IFNs through the formation of immune complexes. Båve et al. (6) have found that the combination of autoantibodies to RNA-binding proteins and material released by apoptotic cells can induce IFNα production by pDCs. This event is probably triggered by the interaction of RNA-containing immune complexes with Fcγ receptor IIa (FCGRIIA) on the surface of pDCs. Lövgren et al. (36) have described the production of IFNα by pDCs stimulated using U1 snRNA combined with IgG from patients with SLE. This response can be inhibited by FCGRIIA antagonists or RNase, suggesting a role for the RNA component as well as FCGRIIA in the immune complex-induced IFNα production by pDCs.

Another role of autoantibodies, especially anti-Ro52, in promoting IFN signaling is based on the function of their target autoantigens. Ro52, also known as tripartite motif-containing protein 21 (TRIM21), is an IFN-inducible E3 ubiquitin-protein ligase that promotes ubiquitination and proteasomal degradation of IRF3 and IRF7 (37, 38). After induction by IFNs following TLR signaling, Ro52 exerts a negative role on IFN signaling and prevents further inflammatory damage. Therefore, autoantibodies against Ro52 may interrupt the negative feedback of type I IFN signaling. Indeed, anti-Ro52 from SS patients is able to inhibit the E3 ligase activity of Ro52 by blocking the E3/E2 interface (39). Additionally, tissue inflammation and systemic autoimmunity in Ro52 knockout mice is thought to be induced by overproduction of pro-inflammatory cytokines (40). These results may well explain the correlation between the IFN signature and autoantibody positivity in SS patients (9). However, anti-Ro alone does not seem sufficient to induce high IFNα activity, given the fact that patients with disease are more likely to have high serum IFN activity than asymptomatic individuals with autoantibodies (41). Therefore, the contribution of autoantibodies to the elevated IFN signaling warrants further study.

## Genetics Risk Factors in IFN Pathways

A possible model for SS development is that an initial viral infection induces the production of type I IFNs and genetic susceptibility factors in certain individuals promote prolonged activation of the IFN system. Genetic predisposition to SS is supported by family aggregation of disease as well as a few twin studies (42–45). Genetic risk variants within or near IFN-regulated genes could possibly predispose patients to increased IFN signaling by (1) the constitutive expression of IFN-inducible genes or (2) the induction of loss-of-function inhibitors within IFN pathways. Genetic studies in SS have relied primarily on candidate gene approaches, focusing on those genes with biological plausibility for a role in SS etiology or evidence of association in other autoimmune diseases (46). The most convincing associations outside the HLA in SS found by candidate gene studies are within the regions of *IRF5* and *STAT4* (47–49), both of which are involved in IFN signaling. IRF5 is a transcription factor mediating type I IFN responses in various immune-related cells (17). Upon viral infection, IRF5 induces the transcription of IFNα and other pro-inflammatory cytokines, including IL12 p40 subunit, IL6, and TNFα (50). Genetic association within the *IRF5* region has been established in other autoimmune diseases, including SLE, RA, ulcerative colitis, primary biliary cirrhosis (PBC), and SSc (51–55). STAT4 is also a critical transcription factor involved in signaling initiated by type I and type II IFNs. It is required for the development of Th1 cells from naive CD4+ T cells and IFNγ production in response to IL12 (56, 57). The association of variants in *STAT4* has also been well established in other inflammatory diseases (58–60). Ongoing genome-wide association studies in SS have firmly established these loci as well as other genes that promote susceptibility to disease and may contribute to the dysregulation of IFN-inducible genes (61).

## Effects of the Overexpression of Type I IFN-Inducible Genes in SS

Type I IFNs are key regulators of human immune systems and exert a broad effect on immune responses and autoimmunity (11). Overexpression of type I IFN-inducible genes in the salivary glands and peripheral blood of SS patients may influence many aspects of SS pathophysiology. Epithelial cells from the salivary glands of SS patients play an active role in promoting immune responses, including increased expression of MHC molecules and co-stimulatory molecules, such as B7 and CD40 (62–65). Many T cell-attracting and germinal center-forming chemokines, such as CXCL10, IL-8, and CXCL13, have also been found to be expressed in epithelial cells from the salivary glands of SS patients (22, 66, 67). Thus, these cells acquire antigen-presenting characteristics, mediating the recruitment, activation, and differentiation of the infiltrating inflammatory cells (68). Many of these molecules are induced by IFNα and IFNβ.

Another cytokine induced by type I IFNs in both salivary gland epithelial cells and peripheral blood monocytes is B cell activating factor (BAFF) (69, 70). BAFF is important in B cell activation, proliferation, and differentiation and has been found to be overexpressed in SS patients (71). Increased expression of BAFF has been observed in salivary gland epithelial cells from SS patients compared with those from healthy controls upon stimulation by IFNα, but not IFNγ or TNFα, suggesting a specific role of type I IFNs in B cell dysfunction in SS (72).

As mentioned above, autoantigen Ro52, or TRIM21 can be induced by IFNα in cultured human B cells and peripheral blood mononuclear cells (73). After upregulation, Ro52 translocates from the cytoplasm to the nucleus and initiates IFNα-induced apoptosis through intrinsic caspase-3. IFNα can also induce the expression of pro-apoptotic molecules, including Fas and FasL (74), and the increased expression of Fas and FasL has been identified in salivary glands from SS patients (18, 75). But this effect is not limited to type I IFNs: IFNγ and TNFα are also potent inducers of these pro-apoptotic molecules in salivary glands (18, 76). Concordantly, elevated levels of epithelial apoptotic cell death have been detected in the minor salivary gland tissues of SS patients (77). In addition, Ro52, Ro60, and La48 redistribute to apoptotic blebs and are exposed to the surface of cells undergoing apoptosis (78). Thus, apoptotic cells are also a main source of the autoantigens that form immune complexes in the salivary gland tissue of SS patients that further boost local IFNα production.

## Conclusion

It is widely acknowledged that SS patients have elevated type I IFN signaling that may contribute to the pathogenesis of this complex disease. Hundreds of genes can be induced by IFNs and are mediated by IFN-related pathways in different cell types, which makes it difficult to attribute disease pathology to the malfunction of a single gene or certain gene family. An efficient approach for unraveling mechanisms of complex diseases is to use multiple tools integrated into systems approaches to answer a specific biological question. High-throughput techniques, such as microarray and genome-wide genotyping arrays, provide us with exceptional opportunities to comprehensively analyze dysregulated IFN networks.

It is worth noting that the IFN signature is only observed in a subset of SS patients who tend to be autoantibody positive (9). This heterogeneity is obviously important to consider and may explain the controversial results of recent IFN-targeting therapies and drug trials for SS (79–83). Eventually the IFN signature in SS may be used as a tool to identify high-risk individuals for preventative strategies, to help diagnose SS through its use as a serum biomarker in lieu of cumbersome and costly routine diagnostic processes currently used in research and clinical practice, and to predict the efficacy of treatment by IFN-related therapies.

## Conflict of Interest Statement

The authors declare that the research was conducted in the absence of any commercial or financial relationships that could be construed as a potential conflict of interest.
